# Genetic changes of non-small cell lung cancer under neoadjuvant therapy

**DOI:** 10.18632/oncotarget.8858

**Published:** 2016-04-20

**Authors:** Arne Warth, Volker Endris, Albrecht Stenzinger, Roland Penzel, Alexander Harms, Thomas Duell, Amir Abdollahi, Michael Lindner, Peter Schirmacher, Thomas Muley, Hendrik Dienemann, Ludger Fink, Alicia Morresi-Hauf, Nicole Pfarr, Wilko Weichert

**Affiliations:** ^1^ Institute of Pathology, Heidelberg University, Heidelberg, Germany; ^2^ Department of Pneumology and Thoracic Oncology, Asklepios Hospital, Munich-Gauting, Germany; ^3^ Department of Radiation Oncology, Heidelberg University, Heidelberg, Germany; ^4^ Department of Thoracic Surgery, Asklepios Hospital, Munich-Gauting, Germany; ^5^ Translational Research Unit, Thoraxklinik at Heidelberg University, Heidelberg, Germany; ^6^ Department of Thoracic Surgery, Thoraxklinik at Heidelberg University, Heidelberg, Germany; ^7^ Institute of Pathology, Wetzlar, Germany; ^8^ Institute of Pathology, Asklepios Hospital, Munich-Gauting, Germany; ^9^ National Center for Tumor Diseases (NCT), Heidelberg, Germany; ^10^ Institute of Pathology, Technical University (TUM), Munich, Germany

**Keywords:** lung cancer, molecular evolution, driver mutations, therapy

## Abstract

**Background:**

Large scale sequencing efforts defined common molecular alterations in non-small cell lung cancer (NSCLC) and revealed potentially druggable mutations. Yet, systematic data on the changes of the respective molecular profiles under standard therapy in NSCLC are limited.

**Results:**

14 out of 68 observed coding mutations (21%) and 6 out of 33 (18%) copy number variations (CNV) were lost or gained during therapy. Mutational and CNV changes clustered in 6/37 (16%) and 3/37 (8%) patients. Changes in clinically relevant mutations were rare but present in single cases for genes such as *BRAF* and *PIK3CA*. The type of radiochemotherapy but not the duration of therapy impacted on the frequency of mutational changes.

**Methods:**

We established a lung cancer specific next-generation sequencing panel covering ~7500 hotspots of 41 genes frequently mutated in NSCLC and performed ultradeep multigene sequencing of 37 corresponding pre- and post-therapeutic formalin fixed paraffin-embedded specimens to discover mutational changes and copy number variations under neo-adjuvant radio- (RTX) and/or chemotherapy (CTX).

**Conclusion:**

We unraveled changes in common driver gene candidates in NSCLC under neo-adjuvant therapy. Our data shed first light on the genetic changes of NSCLC under conventional therapy and might be taken into account when the relevance of sequential biopsy approaches is discussed.

## INTRODUCTION

Molecular characterization of non-small cell lung cancer (NSCLC) with detection of multiple druggable alterations propelled the concept of individualized oncological therapies. Of these, tyrosine kinase inhibitors (TKI) for epidermal growth factor receptor (*EGFR*) mutated or anaplastic lymphoma kinase (*ALK*) translocated NSCLC were already successfully implemented into the clinical setting. In addition, hundreds of novel chemotherapeutic agents are currently tested in clinical trials, many of them in the context of biomarker stratification strategies [1]. However, a major problem of targeted therapies remains the *de novo* existence or development of resistance mechanisms [2–4], leading to an escape of insensitive tumor cell clones or an enrichment of lung cancer stem-like cells [5] and thus tumor progression. Furthermore, intratumoral heterogeneity is considered to foster tumor evolution and adaptation and hinder personalized treatment strategies [6, 7].

Novel next generation sequencing (NGS) methods allow for parallel analyses of genetic aberrations in archived, formalin fixed and paraffin-embedded tissue (FFPE) [8], which is a promising approach to describe genetic changes over time [9]. Such mutational changes might have clinical impact, which is exemplified by the work of Schleiermacher and colleagues who recently demonstrated the emergence of new *ALK* mutations after relapse of neuroblastoma following treatment, some of these mutations were already subclonally present at first diagnosis [10].

In order to shed first light on the molecular alterations of NSCLC under chemo- (CTX) and/or radiotherapy (RTX) we retrospectively analyzed 37 biopsies taken prior to and corresponding resection specimens after neo-adjuvant treatment with a focused NGS approach, covering the most common molecular NSCLC driver mutations.

## RESULTS

### Overall frequency of mutations

**Figure 1:**
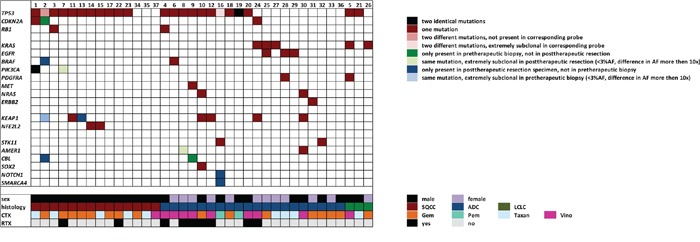
Mutations on a gene by gene and case by case basis shown in relation to clinicopathological and treatment data The first block of genes contains all mutated cell cycle regulators, the second block comprises genes involved in receptor tyrosine kinase signaling, the third block includes genes implicated in xenometabolism and the forth block comprises all other mutated genes.

The most frequently mutated gene in the pre-therapeutic specimens was *TP53* (23 out of 37 cases; 62%, Figure 1, Supplementary Table 1). *TP53* mutations were more frequent in squamous cell carcinoma (SQCC, 11/14 cases, 79%) than in adenocarcinoma (ADC, 10/20 cases, 50%). The second most frequent mutation was found in *KEAP1* (7/37, 19%) with no significant differences between SQCC (3/14, 21%) and ADC (4/20, 20%). *KRAS* mutations were exclusively found in ADC (3/20, 15%) and large cell carcinoma (LC, 2/3, 67%) but not in SQCC. *BRAF* mutations were found in both SQCC (1/14, 7%) and ADC (1/20, 5%). *EGFR* mutations were only found in ADC (3/20, 15%), the same was true for *MET* (1/20, 5%), *NRAS* (2/20, 10%) and *ERBB2* (1/20, 5%) mutations. *PDGFRA* mutations were seen in ADC (1/20, 5%) and LC (1/3, 33%). In contrast, *PIK3CA* mutations were exclusively found in SQCC (2/14, 14%). *CDKN2A* mutations were found in both ADC (1/20, 5%) and SQCC (2/14, 14%), *RB1* mutations were also seen in ADC (1/20, 5%) and SQCC (1/14, 7%). Interestingly, both cases with *RB1* mutation also had a *TP53* mutation raising the question for a potential neuroendocrine differentiation. However, although the mutational spectra were suggestive in this regard, conventional morphology and immunohistochemical analysis against synaptophysin and CD56 did not provide sufficient evidence for a large cell neuroendocrine carcinoma component. *NFE2L2* was only found mutated in 2/14 SQCC (14%). For several other genes only single mutations were noted (Figure 1). None of the cases available for analysis showed ALK protein expression. ROS1 was focally expressed in 2 cases in both corresponding probes but no ROS1 translocations were evident by FISH.

### Mutational gains/losses in pre- and post-therapeutic specimens

**Figure 2:**
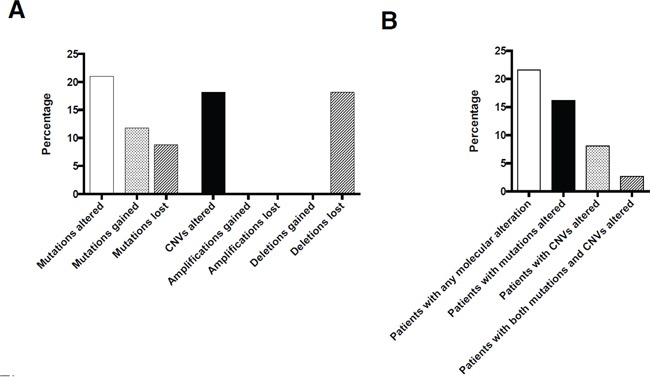
Overall number of molecular changes occurring under therapy on a per mutation/CNV A.and on a per patient basis B

Overall, a total of 68 coding mutations were detected in 19 different genes of pre- and posttherapeutic specimens. First, we investigated whether a mutation was present with an allele frequency above our mutation calling threshold of 3% in both corresponding probes. By doing so, 54/68 mutations (79%) were detected in an allele frequency above the threshold (>3%) in both probes. 14 out of the 68 observed mutations showed overt mutational gains/losses under therapy (21%). Six of these observed molecular alterations were present in the pre-therapeutic biopsies but not in the post-therapeutic resection specimens, eight were present in post-therapeutic resection specimens but not in the pre-therapeutic biopsies (Figures 1, 2).

Secondly, we investigated whether in cases with discordant mutational profiles the corresponding mutations could be detected at all (even at allele frequencies well below 3%) in both probes. In 9 instances the respective molecular alteration was exclusively detected in one of the paired probes (13%) even when the sequencing data of the specific amplicon was reevaluated manually; 3 mutations were only observed in pre-therapeutic biopsies while 6 mutations were only observed in post-therapeutic specimens (Figure 1).

*TP53* mutations were subject to molecular alterations in two instances (Figure 1), however, the loss of one *TP53* mutation was invariably accompanied by the gain of a different *TP53* mutation in both cases, thereby leaving the overall *TP53* mutational status unchanged.

On a case by case basis, molecular alterations clustered in specific probe pairs. 6 out of 37 cases (16%) showed at least one mutational gain/loss (Figure 1), in one case (#2) 6 mutational gains/losses were present.

### Overall frequency of copy number variations

**Figure 3:**
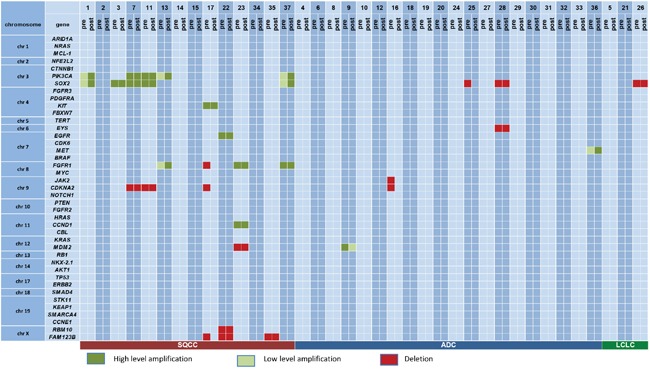
Copy number variations on a case by case basis Genes are sorted according to their chromosomal positions to allow for the assessment of co-deletions/co-amplifications.

The most frequently amplified genes in the pre-therapeutic specimens were *PIK3CA* and *SOX2* (5 out of 37 cases; 13.5%, Figure 3). In four of the cases both genes were coamplified (being both positioned on chromosome 3). Both *SOX2* as well as *PIK3CA* amplifications were only evident in SQCC. The only other gene with recurrent amplifications in our cohort was *FGFR1* with three amplified cases (8%), all these cases were SQCC.

The most frequently deleted gene in pretherapeutic biopsies was *CDKN2A* with 4 cases (11%), three of these cases were SQCC, one was an ADC. *SOX2* deletions were also occasionally observed (3 cases, 8%), exclusively in tumors with non-squamous morphology. Apart from this, only *FAM123B/Amer1* was found recurrently deleted in three cases (8%), all were SQCC.

### Variation of amplifications/deletions in pre-/post-therapeutic specimens

Amplifications, when present, were completely stable in pre- and posttherapeutic specimens. Out of an overall of 18 observed amplifications (clustering in 11 cases) no amplification was gained, none was lost (Figures 2, 3). This was not true for deletions, 6 out of 15 deletions (40%) evident in pretherapeutic biopsies were not detectable in the posttherapeutic resection specimens. The 6 altered deletions clustered in three out of our 37 cases (8%, Figures 3). In contrast, novel deletions post therapy could not be detected in any pair of tumor tissue. Therefore, out of an overall of 33 observed copy number variations (CNV), only 6 showed changes (18%). Out of an overall of 16 patients with CNV three showed alterations (19%).

### Impact of therapy type and duration on molecular profiles

There were no relevant differences in the overall number of molecular alterations for both mutations and CNVs between chemotherapy and chemoradiotherapy. However, when viewed on a per patient basis altered molecular profiles were slightly more likely to occur in patients who received radiochemotherapy when compared to patients who received chemotherapy only.

**Figure 4:**
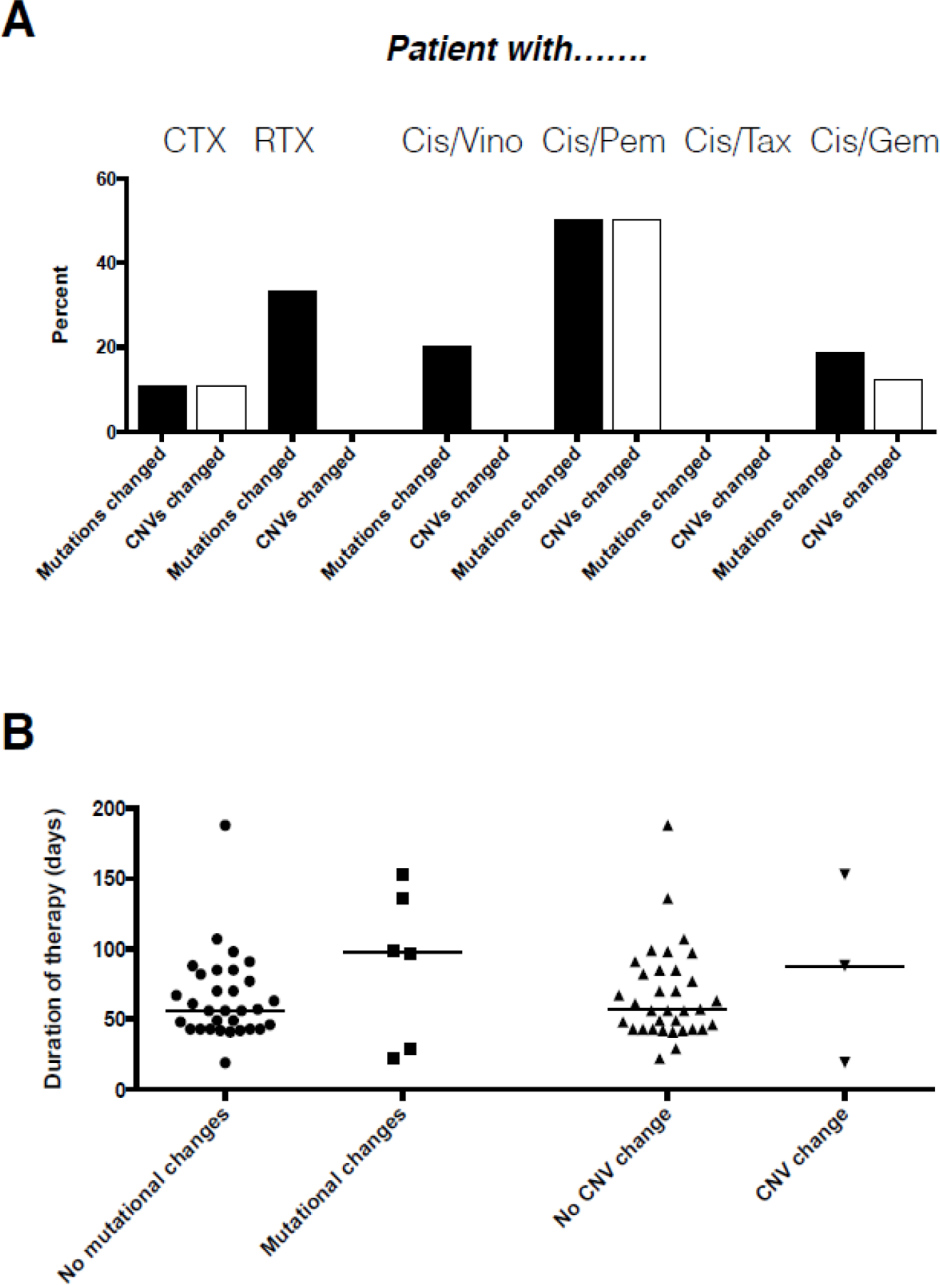
Association of type A. and duration B. of therapy with the occurrence of molecular changes on a per patient basis

The type of chemotherapy administered impacted on the frequency of molecular alterations (Table 1). The combination of taxanes with cisplatin did not induce any molecular alteration. In contrast, gemcitabine and vinorelbine but even more pemetrexed in combination with cisplatin induced substantially higher numbers of molecular alterations, although with only two cases data on the latter combination is only anecdotal. This data was backed when we investigated the association of chemotherapy with the variation of the molecular makeup on a per patient basis (Figure 4). Again, patients receiving taxanes together with platinum had completely stable molecular profiles, while alterations were more frequently observed for vinorelbine, gemcitabine and pemetrexed in combination with cisplatin (Figure 4). However, none of the respective associations reached statistical significancy (p>0.05), likely due to the low number of cases within each group. In contrast to the type of therapy, length of therapy in our setting had no impact whatsoever on the frequency of molecular alterations, both for mutations but also for CNVs (Figure 4).

**Table 1:** Altered mutations/CNVs stratified for therapy administered

	Number of mutations	Number of altered mutations	Number of CNVs	Number of altered CNVs	Overall detected	Overall altered
**RCTX**	20	3 (15%)	7	0 (0%)	27	3 (11%)
**CTX**	48	11 (23%)	26	6 (23%)	74	17 (23%)
**Cis/Vino**	21	2 (10%)	4	0 (0%)	25	2 (8%)
**Cis/Pem**	7	4 (57%)	2	2 (100%)	9	6 (67%)
**Cis/Tax**	14	0 (0%)	9	0 (0%)	23	0 (0%)
**Cis/Gem**	26	8 (31%)	18	4 (22%)	44	12 (27%)

## DISCUSSION

Most NSCLC are detected in advanced, non-resectable tumor stages. Thus, multiple biomarker analyses need to be reliably performed using sparse biopsy or even cytology material. Focused NGS approaches are a time- and cost-effective strategy to face these challenges. After establishment of a respective multigene test for NSCLC using FFPE material [8], we have implemented this type of analysis into routine diagnostics. Here, we applied this technology to sequentially obtained specimens of NSCLC patients. The reliable performance of this approach is mirrored by both the detection rates of common mutations and CNVs but also by the detection of entity specific mutations and CNVs exclusively in ADC or SQCC, largely in accordance with data from previous studies [11–14, 18, 20, 21].

Over the last decade lung cancer diagnostics and therapy emerged as a prime example of individualized medicine with testing for tissue-derived biomarkers as a prerequisite for tumor-specific therapies. However, clonal tumor evolution and progression due to the development of resistance mechanisms challenges the concept of single biomarker analysis as well as biomarker analysis using only archived tumor material obtained month or years prior to therapy. Concerning the potential heterogeneity of molecular alterations between NSCLC primary and synchronous metastases a most recent study revealed a high concordance of recurrent somatic alterations [19], pointing out that resistance mechanisms rather develop over time in response to therapy and might usually not be *a priori* detectable at different (including metastatic) tumor sites. In order to adapt biomarker-driven treatment concepts to the most current tumor biology and to develop respective combined treatment approaches targeting resistance mechanisms after tumor progression, it is important to specify the underlying genetic changes of molecular tumor profiles over time and in response to widely administered RTX/CTX. We show that such changes occur under conventional therapies, but only to a minor extent both for a selected set of tumor relevant mutations and also for CNVs.

About one fifth of the detected mutations showed overt gains/losses under therapy with a slight propensity towards increased mutational rates and decreased CNV rates in the post-therapeutic specimens. The overall quite high stability of mutational profiles under therapy is noteworthy and was most recently also described in a series of 50 matched NSCLC pairs post therapy which were analyzed for *EGFR, KRAS, BRAF, PIK3CA, HER2, ALK*, and *MET* alterations [22]. Nevertheless, although molecular changes are infrequent and cluster in a minority of patients, we showed that even over the limited period of time defined by neoadjuvant therapy occasionally therapy relevant molecular alterations in genes such as *BRAF* do occur.

Ionizing radiation produces rearrangements of the genome resulting in chromosomal instability, in which new aberrations continue to arise even many generations after irradiation. The underlying mechanisms are described by a breakage-and-reunion model where non-homologous end-joining of radiogenic DNA double strand breaks is considered the dominant recombinational mechanism during cell cycle [23]. In addition, analyses of lymphocytes from patients with various malignancies including solid tumors treated with CTX/RTX and respective controls revealed that the geometric mean mutation frequency of the lymphocytes in untreated patients was 6.72 × 10^−6^, which was significantly increased to 19.57 × 10^−6^ following CHX and 34.40 × 10^−6^ following RTX/CHX [24]. However, in our experimental approach clear cut differences in the evolution of mutational profiles between CTX and RCTX were not evident within our limited observational period.

In contrast, platinum-based regimens with gemcitabine, pemetrexed or vinorelbine were associated with higher rates of molecular alterations than the combination with taxanes. This might point to the fact that therapy regimens including taxanes are potentially more effectively suppressing the generation of novel clones and thereby the occurrence of resistance. However, to unravel the underlying mechanisms and affected pathways which are altered by specific regimens and to elucidate how to adopt more effective treatments to the dynamic changes of molecular profiles, clearly more and large scale studies of sequential biopsies compiled by large consortial efforts [25] are required. The spread of somatic mutations in a tumor is complex and influenced by multiple factors, for example the mutation rate, the number of cell divisions, the nature of competition between different cellular lineages, but also different tissue architectures. Even more complicating, cancer progression is affected by many additional processes including responses of the immune system, hormonal status, gene expression, and signaling between tissues [26] as well as complex interplays between tumor suppressors, oncogenes, and genetic instability [27]. These variables need to be assessed in integrated analyses to ultimately understand tumor escape mechanisms associated with specific therapeutic modalities and to develop effective strategies against the occurring of resistance.

Limitations of our study include the comparably low number, but also a certain degree of heterogeneity of the analyzed cases concerning NSCLC subtype, treatment modality, duration of treatment, and interval between biopsy and resection. However, one has to take into account that it is extremely difficult to obtain such paired specimens in the lung cancer setting at all. Moreover, our cohort largely recapitulates the real world routine diagnostic setting, where pathologists and clinicians are also faced with this high degree of heterogeneity. In addition, in the herein chosen approach we only analyzed selected, frequently altered lung cancer relevant genes with supposed clinical and tumorbiological relevance, which does not allow for the detection of “new” candidate genes emerging under therapy. Subsequent large scale studies [25] will have to extent our data with respect to novel candidates involved in therapy-dependent genetic changes of NSCLC. In addition, since low tumor cell content might also impact on sequencing results in our experimental setup, we aimed at a mean amplicon coverage of well above 3000 fold and we did not include cases with a tumor cell content below 10% in our study cohort (see Supplementary Table 1), however we cannot exclude that these variables have also impacted on our results in some instances. And finally, with our experimental approach using pretherapeutic biopsies as the templates to assess the initial molecular makeup we are not able to reliably discern upfront molecular tumor heterogeneity from tumor evolution, however, like in real world diagnostic obviously this limitation is not avoidable in our experimental setup.

In conclusion, our study sheds first light on the dynamic changes of common and clinically relevant genetic profiles of non-small cell lung cancer under conventional radio-/chemotherapeutic treatment. We demonstrate that losses/gains of mutations and CNVs occur in a substantial but still minor number of cases and that the number of changes seems to be therapy type but not therapy duration dependent. These data might be taken into account when strategies for sequential biopsy approaches for biomarker stratification are issued.

## MATERIALS AND METHODS

### Cohort

We screened the clinical files as well as tissue archives of three large German lung clinics (Heidelberg University, Asklepios Hospital Munich-Gauting, Wetzlar) for available tumor tissues of NSCLC patients treated with neo-adjuvant therapy. Usage of the tissue was approved by the local ethics committees.

In the analyzed cohort (n=37) 25 patients (68%) were male and 12 were female (32%). Mean age at the time of diagnosis was 60.6 years (range: 36-77 years). 20 patients (54%) had ADC, 14 (38%) had SQCC, and three (8%) had LC. 28 patients received neo-adjuvant CTX (76%), 9 patients received neo-adjuvant CTX/RTX (24%). Median time between initial biopsy and resection was 111 days (quartiles: 80.5, 136.5 days), median duration of therapy was 57 days (quartiles 43, 85.5 days). 16 (43%) patients received platinum (either cis-/or carboplatinum) plus Gemcitabine, 10 (27%) received platinum plus Vinorelbine, 9 (24%) received platinum plus Taxanes, 2 (6%) received platinum plus Pemetrexed (Figure 1).

### Tumor material and DNA extraction

Tumor areas were marked on an H&E slide by an experienced pulmonary pathologist (AW) and the tumor cell content was determined by estimating the percentage of neoplastic and non-neoplastic cells in the areas marked (Supplementary Table 1). Corresponding tissue areas were microdissected from subsequent unstained slides. Extraction of genomic DNA was performed after proteinase K digestion by fully automated purification using the Maxwell16 Research System (Promega, Madison, USA). DNA content was measured fluorimetrically using the QuBit HS DNA Assay (Thermo Fisher Scientific, Waltham, USA) and DNA sequencing grade quality was confirmed using a real-time qPCR-based method (RNAseP Detection system, Thermo Fisher Scientific, Waltham, USA).

### Panel design

TCGA datasets, the COSMIC database as well as data from recent publications [11–14] [15] were evaluated and genes which have been reported as being frequently mutated were included in our panel design. As in every focused multigene sequencing approach, we had to omit some potentially relevant genes lacking a defined mutational hotspot and/or comprising a large number of amplicons. Using the mutated loci as input, we constructed a lung cancer panel using the Ion AmpliSeq Designer (Thermo Fisher Scientific, Waltham, USA). Our final panel included 139 amplicons covering hotspot regions of 41 genes (Supplementary Table 2).

### Library preparation and semiconductor sequencing

For library preparation, the multiplex PCR-based Ion Torrent AmpliSeq™ technology (Thermo Fisher Scientific, Waltham, USA) with our custom designed panel was used. Amplicon library preparation was performed with the Ion AmpliSeq Library Kit v2.0 using 10 ng of DNA. DNA was mixed with primer pools, containing all primers for generating the 139 amplicons and the AmpliSeq HiFi Master Mix and transferred to a PCR cycler (Biometra, Goettingen, Germany). Subsequent to PCR, primer end sequences were partially digested using FuPa reagent, followed by ligation of barcoded sequencing adapters (Ion Xpress Barcode Adapters 1-16, Thermo Fisher Scientific, Waltham, USA). The final library was purified using AMPure XP magnetic beads (Beckman Coulter, Krefeld, Germany) and quantified using qPCR (Ion Library Quantitation Kit) on a StepOne*Plus* qPCR machine (both Thermo Fisher Scientific, Waltham, USA). The individual libraries were diluted to a final concentration of 100 pM and eight to ten libraries were pooled and processed to library amplification on Ion Spheres using Ion PGM™ Template OT2 200 Kit (Thermo Fisher Scientific, Waltham, USA). Non-enriched libraries were quality-controlled using Ion Sphere quality control measurement on a QuBit instrument. After library enrichment (Ion OneTouch ES), the library was processed for sequencing using the IonTorrent 200 bp sequencing v2 chemistry and the barcoded libraries were loaded onto a chip. Pooling of eight samples on a 318v2 chip resulted in a mean coverage of 3000 fold per amplicon.

### Variant calling and annotation

Data analyses were performed using IonTorrent Suite Software (version 4.4.3). After base calling, reads were aligned against the human genome (hg19) using the TMAP algorithm. Variant calling was performed with the build-in variant caller plugin using a corresponding bed-file containing the coordinates of the amplified regions and comprising also indels with up to 30 bases. In principle, only variants with an allele frequency >3% and a minimum coverage >200 reads were taken into account. However, if one of the samples contained a specific mutation, which met these criteria, the corresponding sample was rechecked for the presence/absence of this mutation, regardless of allele frequencies. Variant annotation was performed using the CLC Genomics Workbench (version 8.0.2). Annotations included information about nucleotide and amino acid changes of RefSeq annotated genes, COSMIC and dbSNP entries as well as detection of possible splice site mutations. For data interpretation and verification, the aligned reads were visualized using the IGV browser (Broad Institute) [16]. Only non-synonymous single nucleotide polymorphisms (SNP) were considered. SNPs listed in the 6500Exome database and/or dbSNP were excluded. Furthermore SNPs without any annotation were excluded if their allele frequency approached 100%, since we assume that these alterations where of germline origin.

### Prediction of copy number alterations

Copy number variations (CNVs; amplifications and deletions) were identified by using the coverage data summary for each sample and each amplicon generated by the TorrentSuite software. Detection of CNVs was performed according to Endris et al. (2013) [8]. In brief, gene amplifications and/or deletions were determined by a simple algorithm using the number of reads of each individual amplicon in the sequenced sample: i) dividing number of reads of each individual pool-amplicon by the total number of sequencing reads of the respective sample = (reads amplicon x/total reads) = normalized amplicon read depth value (NARD), ii) Multiplication of NARD by total number of amplicons (e.g. Lung cancer panel = 140 amplicons; NARD (reads amplicon x/total reads) × 140), iii) determination of median normalized amplicon read depth (MNARD) of all samples = median (NARDsample1:NARDsamplex), reflecting the typical amplification efficiency of each individual amplicon in the pool, and iv) determination of the standard deviation (SD) from the median value. Amplifications are considered as true if the NARDs of all amplicons covering a gene differ by >2 SD from the median value. On the other side, deletions are considered as true if the SD of all amplicons covering a gene is <0.5.

### Immunohistochemistry and fluorescence *in situ* hybridization

Sufficient tumor material for further ALK and ROS1 immunohistochemistry was available from 16 tumor pairs. To address potential translocations of ALK or ROS1 the respective protein expression was analyzed by immunohistochemistry, positive cases were further analysed by FISH as described previously [17, 18].

### Statistical analysis

Statistical analyses were carried out with SPSS 20 (IBM, Armonk, USA) and GraphPad Prism 4 (GraphPad Software, La Jolla, USA). Significance of correlations between molecular alterations and clinicopathological data was tested by χ2 test, χ2 test for trends and Mann-Whitney as well as Kruskal-Wallis test. P-values <0.05 were considered statistically significant.

## SUPPLEMENTARY TABLES






