# Supplementary material for: Genetic changes of non-small cell lung cancer under neoadjuvant therapy
Source: Oncotarget. 2016 Apr 20;7(20):29761–9. doi: 10.18632/oncotarget.8858 (PMC5045431; doi:10.18632/oncotarget.8858)
Supplement: Supplementary file 1 [file oncotarget-07-29761-s001.pdf]

Genetic changes of non-small cell lung cancer under neo-adjuvant therapy

SUPPLEMENTARY TABLES

Supplementary Table S1: Overview of all molecular alterations on a case by case basis

See Supplementary File 1

Supplementary Table S2: Exons and genes covered by our customized lung cancer next-generation sequencing panel

See Supplementary File 2
