# Supplementary material for: Genetic changes of non-small cell lung cancer under neoadjuvant therapy
Source: Oncotarget. 2016 Apr 20;7(20):29761–9. doi: 10.18632/oncotarget.8858 (PMC5045431; doi:10.18632/oncotarget.8858)
Supplement: Supplementary file 2 [file oncotarget-07-29761-s003.docx]

**ARID1A**	2, 3, 10, 11, 14, 17, 20	**KIT**	9, 11	**MYC**	2, 3	**KRAS**	2, 3	**KEAP1**	2, 3, 4, 5
**NRAS**	2, 3	**FBXW7**	9, 10, 11	**JAK2**	12, 14, 25	**MDM2**	4, 11	**SMARCA4**	22, 23, 25, 26
**MCL-1**	1, 3	**TERT**	2, 11	**CDKNA2**	1, 2	**RB1**	2, 3, 6, 13, 16, 17, 18, 20, 21, 22, 23	**CCNE1**	5, 10
**NFE2L2**	2	**EYS**	5, 7	**NOTCH1**	6, 8, 18, 22	**NKX-2.1**	1, 2	**RBM10**	3, 5
**CTNNB1**	3, 5, 9	**EGFR**	18, 19, 20, 21	**PTEN**	1, 3, 5, 6, 7, 8	**AKT1**	3	**FAM123B**	1
**PIK3CA**	2, 5, 8, 10, 14, 21	**CDK6**	3, 5	**FGFR2**	7, 13	**TP53**	4, 5, 6, 7, 8, 9, 10		
**SOX2**	1	**MET**	14, 16	**HRAS**	2, 3	**ERBB2**	19, 20		
**FGFR3**	7, 16	**BRAF**	11, 15	**CCND1**	1, 3	**SMAD4**	5, 7, 9, 12		
**PDGFRA**	5, 10, 11, 12, 18, 22	**FGFR1**	7, 10	**CBL**	2, 8, 9	**STK11**	1, 2, 4, 5, 6, 8		
